# Alterations in the *Helicoverpa armigera* Midgut Digestive Physiology after Ingestion of Pigeon Pea Inducible Leucine Aminopeptidase

**DOI:** 10.1371/journal.pone.0074889

**Published:** 2013-09-30

**Authors:** Purushottam R. Lomate, Bhakti R. Jadhav, Ashok P. Giri, Vandana K. Hivrale

**Affiliations:** 1 Department of Biochemistry, Dr. Babasaheb Ambedkar Marathwada University, Aurangabad, Maharashtra State, India; 2 Plant Molecular Biology Unit, Division of Biochemical Sciences, CSIR-National Chemical Laboratory, Pune, Maharashtra State, India; University of Tennessee, United States of America

## Abstract

Jasmonate inducible plant leucine aminopeptidase (LAP) is proposed to serve as direct defense in the insect midgut. However, exact functions of inducible plant LAPs in the insect midgut remain to be estimated. In the present investigation, we report the direct defensive role of pigeon pea inducible LAP in the midgut of *Helicoverpa armigera* (Lepidoptera: Noctuidae) and responses of midgut soluble aminopeptidases and serine proteinases upon LAP ingestion. Larval growth and survival was significantly reduced on the diets supplemented with pigeon pea LAP. Aminopeptidase activities in larvae remain unaltered in presence or absence of inducible LAP in the diet. On the contrary, serine proteinase activities were significantly decreased in the larvae reared on pigeon pea LAP containing diet as compared to larvae fed on diet without LAP. Our data suggest that pigeon pea inducible LAP is responsible for the degradation of midgut serine proteinases upon ingestion. Reduction in the aminopeptidase activity with L*p*NA in the *H. armigera* larvae was compensated with an induction of aminopeptidase activity with A*p*NA. Our findings could be helpful to further dissect the roles of plant inducible LAPs in the direct plant defense against herbivory.

## Introduction

Plants have evolved several defensive mechanisms to cope up with the threat of phytophagous insects [1], [2]. At the same time insects have also evolved mechanisms to detoxify the plant defensive compounds. Digestive proteolytic activity in the herbivore gut might be the key in the adaptation of insects against plant defensive chemicals [3], [4]. It is now well known that along with proteinase inhibitors (PIs), some plant enzymes also induce after insect attack, mechanical wounding and external applications of jasmonic acid (JA) or methyl jasmonate (MeJA) [5]–[10]. Decreased growth of herbivores on plants cannot be explained solely by the action of PIs but rather involves multiple host compounds that exert a combination of toxic, antinutritive, and antifeedent effects [11], [12]. Jasmonate and wound inducible plant enzymes work together with PIs to reduce the growth of herbivory [9]. Chen *et al.,* [9] studied the role of jasmonate inducible plant enzymes in the insect gut. Plant arginase and threonine deaminase remained active in the midgut of *Manduca sexta* and found to be involved in the catabolism of essential amino acids Arg and Thr, respectively [9].

Similarly jasmonate and wound inducible leucine aminopeptidases (LAPs) are one of the most studied plant defensive enzymes which are supposed to serve a defensive role in the lepidopteran gut [13], [14]. Although plant LAPs have been intensively studied and characterized especially in tomato (*Solanum lycopersicum*), their function and role in defense are still not completely understood [15]. Walling [15] postulated a regulatory role for LAP in the wound signaling cascade; others have hypothesized that LAP has a direct antinutritive effect in insect guts [9], [13]. In *Solanum nigrum* virus induced gene silencing of LAP proved its defensive function against herbivores [16]. It was reported that LAP-A has a role in modulating defenses against herbivores by promoting late wound responses and acting downstream of JA biosynthesis and perception [17]. Earlier, we have been reported the induction of LAP-like enzyme in non-solanaceous plant namely pigeon pea (*Cajanas cajan*) after wounding and application of MeJA [18]. LAP-A was among the most abundant jasmonate inducible proteins identified in the midgut of *M. sexta* larvae grown on induced plants [9]. Although it remains to be determined whether LAP-A disrupts digestive physiology, it is notable that the enzyme efficiently liberates Arg (in addition to leucine) from the N-terminal end of polypeptide [14]. Thus, it might be possible that LAP-A and arginase act synergistically in the gut to catabolize protein derived Arg, which is an important determinant for optimal growth of leaf eating insects [11]. However, exact role of LAPs in insect gut needs to be estimated.

Altogether, it seems that aminopeptidases are crucial enzymes in plant-insect interactions. In this scenario, it is essential to check the changes in insect digestive physiology and responses of midgut aminopeptidase and serine proteinases after ingestion of plant inducible LAPs. In the present paper we have tried to explore the same. Earlier we have characterized jasmonate inducible aminopeptidase from pigeon pea [18] and soluble aminopeptidases from the midgut of *H. armigera* [19]. Here, we report the effects of pigeon pea inducible aminopeptidase in the midgut of *H. armigera*. It would be interesting to understand the direct defensive role and interactions of plant inducible aminopeptidase in the insect midgut with serine proteinases and aminopeptidases.

## Material and Methods

### Chemicals

The following chemicals used for experiments were obtained from Sigma-Aldrich, St. Louis, MO, USA: leucine *p*-nitroanilide (L*p*NA), alanyl *p*-nitroanilide (A*p*NA), valine *p*-nitroanilide (V*p*NA), N-α benzoyl-DL-arginine *p*-nitroanilide (BA*p*NA), N-succinyl-Ala-Ala-Pro-Phe *p*-nitroanilide (SAAPF*p*NA), gelatin and bovine serum albumin (BSA). Chemicals for electrophoresis were purchased from Merck, Darmstadt, Germany and Sisco Research Laboratory, Mumbai, MS, India. All other chemicals used were of high analytical grade.

### Plant Material

**Figure 1:**
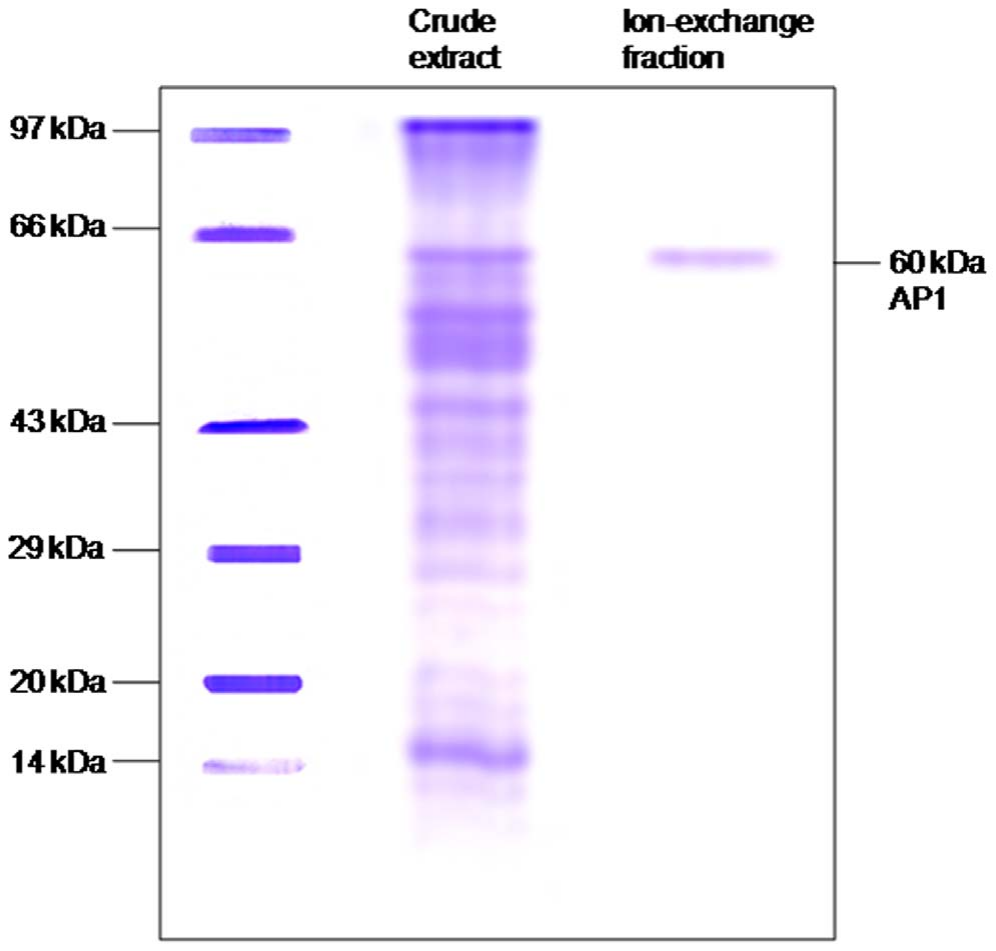
Protein SDS-PAGE of purified inducible LAP from pigeon pea leaves. The crude leaf extract and ion-exchange fraction were loaded on 12% SDS-PAGE and separated protein bands were visualized by staining with CBBR-250. Lane 1, Molecular weight markers, Lane 2, crude leaf extract and Lane 3, purified LAP from ion-exchange fraction.

Pigeon pea seeds were germinated for 24 h in sprout maker and seedlings were transferred to pots. Plants were grown in glasshouse (14 h light: 10 h dark, 35 to 40°C light: 20 to 25°C dark; 50 to 60% relative humidity). MeJA treatment: a 20 mL quantity of lanoline was poured into centrifuge tubes, 200 mL of MeJA was added, and the mixture was rapidly stirred. The mixture was poured into petri-plates, plates were sealed and mixture was allowed to solidify and applied to the stem or abaxial surface of the midrib of a leaf of ∼ 3 month old plants by needle; control plants were kept aside. Treated and untreated leaves were harvested and inducible aminopeptidase was electrophoretically detected. The purified inducible aminopeptidase was used for insect feeding assays. First step of LAP purification was performed as described earlier [18] and further using additional two steps of gel filtration and one step of ion-exchange chromatography. Aminopeptidase activity fractions obtained from gel filtration chromatography were concentrated with acetone precipitation and loaded on DEAE-cellulose anion-exchange column (2.5×23 cm) equilibrated with 0.1 M Tris–HCl pH 8.0. Linearly increasing salt gradient (0 to 1 M NaCl in 0.01 M Tris–HCl pH 8.0) was used for elution. Fractions of 2 mL were collected and protein concentration was determined as described by Lowry et al., [20]. Aminopeptidase activity from eluted fractions was determined using L*p*NA as substrate. The assay was started by adding eluted fraction (40 µg of proteins each) in 200 µL of 10 mM L*p*NA prepared in 0.1 M Tris-HCl buffer pH 8.0 and continued up to 30 min at 37°C. After 30 min the reactions were terminated by adding 150 µL of 30% acetic acid. The rate of production of *p*-nitroaniline was measured at 410 nm. The crude leaf extract and final activity fraction eluted through ion exchange column were loaded on 12% SDS-PAGE and the electrophoresis was carried out. After electrophoretic run the gel was stained with CBBR-250 to visualize the protein bands. The results of SDS-PAGE are presented in the Figure 1.

### Insect Culture and Feeding Assays

*H. armigera* larvae were obtained from International Crop Research Institute for Semi Arid Tropics (ICRISAT) Patancheru, Hyderabad, India and maintained at 27±2°C, 60±5% relative humidity. The artificial diet (AD) was prepared as described by Nagarkatti and Prakash [21]. Larvae were maintained under laboratory conditions on an artificial diet and subsequent generations were used for feeding assays.

Newly emerged larvae were fed on artificial diet or diet supplemented with pigeon pea inducible LAP. The major ingredients of the control diet were sorbic acid, ascorbic acid, methyl *p*-hydroxy benzoate, vitamin B-complex and defatted chickpea powder. LAP-diet was prepared by incorporating LAP into the artificial diet (pigeon pea LAP (10 µg/g of diet) (specific activity 721 µM/mg/min). The LAP activity assay was carried out after preparation of diet to check LAP stability and activity. The LAP denatured by heating was used as alternative control. Thirty neonates were transferred to control as well as test diets. Three set containing thirty neonates each were maintained on respective diet as replicates (i.e. total 90 insects each on control and test diet and the assay was repeated three times). Fresh diets were fed daily in individual vials containing one larva. Insects were maintained at 28±2°C, 60% RH and a photoperiod of 14 h light and 10 h dark. The feeding assays were continued up to 15 d and the larval masses were recorded. The early 5^th^ instar actively feeding larvae from each diet were selected for further experiments. All the larvae fed on each above mentioned diets were immobilized by keeping at −20°C for 30 min, dissected ventrally and midguts were removed. Midgut extracts of larvae fed on each diet were pooled separately, frozen and stored at −20°C until further use.

### Preparation of Midgut Extracts

Midgut tissues of the larvae fed on each above mentioned diets were removed, weighed and homogenized with a pre-chilled mortar and pestle in 1∶6 (w/v) volume of ice-cold 0.1 M Tris-HCl buffer pH 8.0. Homogenates were centrifuged at 10,000 g at 4°C for 20 min. The supernatants were collected and divided into small aliquots and stored at −20°C until use. Protein concentration in supernatants was estimated by the method of Lowry using bovine serum albumin as standard [20].

### In-gel Visualization of Aminopeptidase Isoforms

Aminopeptidase isoforms were separated on native PAGE (4% stacking gel and 8% resolving gel) [22] and detected by Zymographic analysis [19], [23]. Midgut extracts (100 µg of proteins each) of larvae fed on diet with LAP or without LAP were loaded on native PAGE and electrophoresis was carried out at constant current of 20 mA. After electrophoresis, the gel was washed twice with distilled water for 10 min and equilibrated with 0.1 M Tris-HCl buffer pH 8.0, twice for 5 min. After that, the gel was incubated into 10 mM L*p*NA solution prepared in 0.1 M Tris-HCl buffer pH 8.0 (L*p*NA was initially solubilized in 800 µL of dimethyl sulphoxide and further diluted to 10 mL with buffer) and incubated at 37°C for 20 min. Diazotization of liberated *p*-nitroaniline was performed at 0°C by immersing the gel into freshly made 0.1% (w/v) sodium nitrite solution in 1 M HCl for 2 min. The excess sodium nitrite was removed with 1% (w/v) urea, the reaction being continued for 30 s by gentle shaking of the gel. The diazotized gel then immersed into 0.025% (w/v) 1-naphthylamine solution in 22% (v/v) ethanol with gentle agitation until distinct pink azo dye formed (2–5 min).

### Visualization of Proteinase Isoforms

Proteinase isoforms in the midgut extracts were electrophoretically separated on discontinuous native PAGE (4% stacking and 8% resolving gel) and visualized by using gelatin reverse zymography [24]. Midgut extracts (40 µg of proteins each) of larvae fed on diet with LAP or without LAP were separated on 8% native PAGE. After electrophoresis the gel was washed with distilled water and equilibrated in 0.1 M Glycine-NaOH buffer pH 9.6. After equilibration the gel was placed in 1% (w/v) gelatin solution (prepared in the same buffer) and incubated for 1 h at 37°C. Then the gel was stained with CBBR-250 and destained to visualize proteinase activity isoforms.

### Aminopeptidase, Trypsin and Chymotrypsin Activity Assays

Midgut extracts of larvae fed on diet with LAP or without LAP were used for aminopeptidase activity assays. Respective reactions were started by adding each midgut extract (40 µg of proteins each) in 200 µL of 10 mM L*p*NA prepared in 0.1 M Tris-HCl buffer pH 8.0 and continued up to 30 min at 37°C. After 30 min the reactions were terminated by adding 150 µL of 30% acetic acid. The rate of production of *p*-nitroaniline was measured at 410 nm. Trypsin and chymotrypsin activity assays were carried out using selective substrates. Midgut extracts of larvae fed diet with LAP or without LAP were used for the assay of trypsin and chymotrypsin activity. Respective reactions were started by adding each midgut extract (40 µg of proteins each) in 200 µL of 10 mM BA*p*NA (specific substrate for trypsin like enzymes) or SAAPF*p*NA (specific substrate for chymotrypsin like enzymes) prepared in 0.1 M Glycine-NaOH buffer pH 9.6 (substrates were initially solubilized in 800 µL dimethyl sulphoxide or dimethyl formamide and further diluted to 10 mL with buffer) and continued up to 1 h at 37°C. After 1 h the reactions were terminated by adding 150 µL of 30% acetic acid. The rate of production of *p*-nitroaniline was measured at 410 nm.

### Interactions of Pigeon Pea Inducible LAP with *H.* *armigera* Gut Proteinases (HGPs)

Pigeon pea LAP (40 µg protein) and midgut extract (40 µg protein) of larvae fed on control diet were pre-incubated at 37°C for 30 min. The mixture was loaded on 8% native PAGE and aminopeptidase activity isoforms were visualized as mentioned in the above method. The mixture was also processed for the estimation of aminopeptidase activity. Aminopeptidase activity was determined using L*p*NA as substrate.

Pigeon pea LAP (40 µg protein) and midgut extract (40 µg protein) of larvae fed on control diet were pre-incubated at 37°C for 30 min. The mixture was loaded on 8% native PAGE and proteinase activity isoforms were visualized by gelatin reverse zymography as mentioned in the above method. The mixture was also processed for the estimation of trypsin and chymotrypsin activities.

### Substrate Specificities of Aminopeptidases

Substrate specificities of midgut aminopeptidases of larvae fed on control and test diets were investigated using three synthetic substrates for aminopeptidases namely L*p*NA, A*p*NA and V*p*NA. In all reactions, reaction rate was linear with time, protein content and independent of substrate concentration. An assay was carried out as described earlier for L*p*NA.

### Statistical Analysis

All the experiments were carried out at least three times with three replications. The data were subjected to analysis of variance and post hoc tests to compare the significance of differences at *p*<0.05. The statistical analysis was performed using SPSS 15·0 (SPSS Inc., Chicago, IL, USA).

## Results

### Induced Pigeon Pea LAP causes Adverse Effect on *H. armigera* Larvae

**Figure 2:**
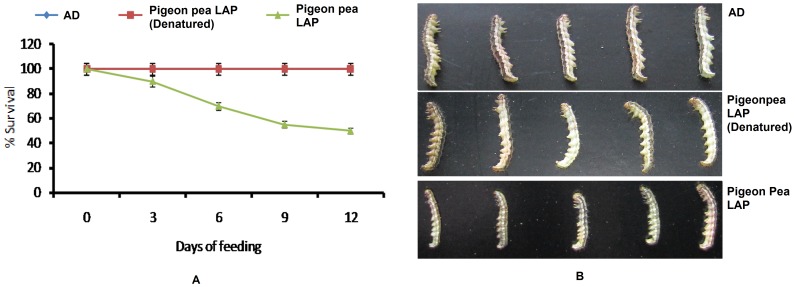
Growth and survival rate of *H. armigera* larvae on artificial diet and diet containing pigeon pea inducible LAP. A- Survival rate of *Helicoverpa armigera* on a control diet versus test diet containing pigeon pea inducible LAP. The neonates were fed upon diet containing pigeon pea inducible LAP. The feeding analysis was carried out till 12 days and rate of survival was recorded every 3 day. Data were analyzed by a nonparametric chi-square test to determine significant differences in the percentage survival of *H. armigera* fed on the test diet (*n* = 30 individuals per test, three replicates). The highest survival rate was recorded on the control diet, whereas the lowest survival rate was recorded on the diet containing plant LAP. B- Development of *H. armigera* fed on control and test diet. Larvae fed on control diet showing normal growth, while larvae fed on plant LAP showing retarded or stunted growth. The aminopeptidase denatured by heating was used as+ve control on which the larval growth was found similar to as of control.

**Figure 3:**
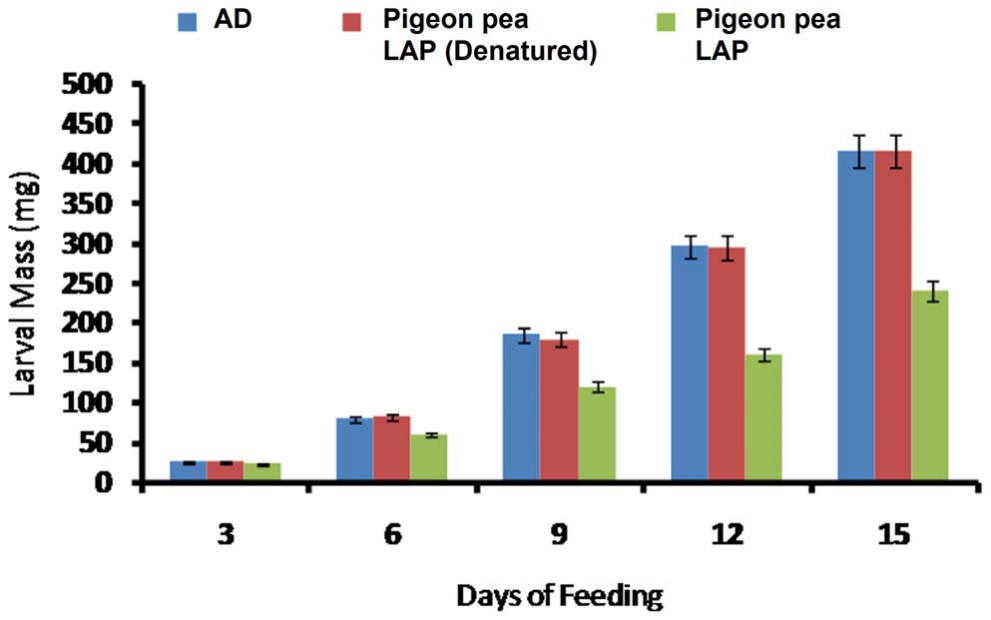
Evaluation of larval mass of *H. armigera* larvae fed on control diet and diet containing plant LAP and denatured plant LAP. The experiment was performed in three replicates, and each replicate contains thirty larvae. Significant differences in larval mass were calculated by Student *t* tests. Error bars indicate ± SD. Significant difference in the mass of larvae fed on control and test diet was observed on day 9 (*P*<0.01) and days 12 and 15 (*P*<0.001) of feeding. Larval mass gain was adversely affected to feeding on inducible LAP.

The *H. armigera* larvae were fed on diet with LAP or without LAP incorporated with pigeon pea inducible LAP. The results of the feeding bioassays showed that 100% survival was recorded for *H. armigera* fed on diet without LAP as well as with LAP up to third day. However, significant differences were recorded among the treatments in terms of percentage survival on day 6 (χ2 = 16.8, d.f. = 2, *P<*0.01), 9 and 12 (χ2 = 102, d.f. = 2, *P<*0.01) days of feeding. As expected, the highest survival rate was recorded on the diet without LAP, whereas the lowest survival rate was recorded on the diet containing pigeon pea LAP (Figure 2A). Further, the *H. armigera* larvae reared on diet without LAP showed the normal growth whereas the larvae reared on diet containing pigeon pea LAP showed stunted growth (Figure 2B). However, significant difference between the masses of larvae fed on LAP-diet and without LAP was observed on day 9 (*P*<0.01) and days 12 and 15 (*P*<0.001) of feeding. Larval mass gain was adversely affected to feeding on inducible LAP (Figure 3).

### Differential Accumulation of Proteinase Activity in *H. armigera* in Response to LAP

**Figure 4:**
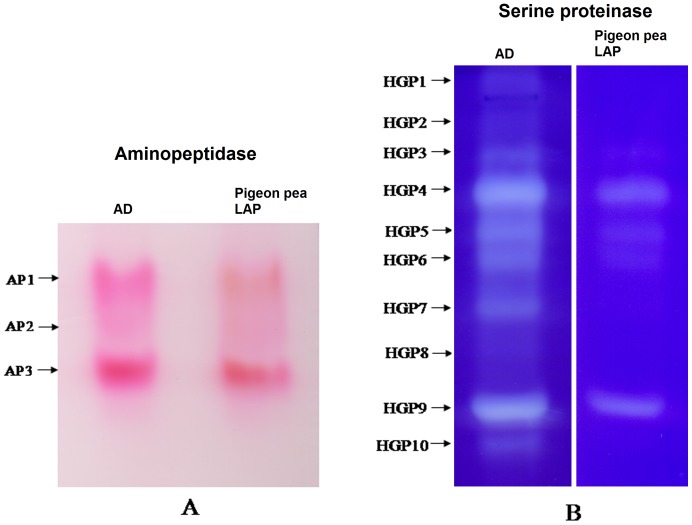
In-gel visualization of aminopeptidase and proteinase activity isoforms in the midgut of *H. armigera* larvae fed on artificial diet and diet incorporated with plant LAP. A- Midgut extracts (100 µg of proteins each) were resolved on 8% native discontinuous polyacrylamide gels and aminopeptidase activities were stained with 1- naphthylamine by diazotizing the released *p*-nitroaniline from L*p*NA with sodium nitrite. Three pink bands of the aminopeptidase activity were observed and these bands were designated as AP1 to AP3. No difference in aminopeptidase activity band pattern was observed between larvae fed on artificial diet and diet supplemented with LAP. B- Midgut extracts (40 µg of proteins each) were resolved on 8% native discontinuous polyacrylamide gels and proteinase activity isoforms were detected using gelatin reverse zymography. The proteinase activity bands were observed white against blue background. These bands were designated as HGP1 to HGP10. The activity of proteinase isoforms was significantly decreased in the midgut extracts of larvae reared on diet supplemented with pigeon pea LAP.

**Figure 5:**
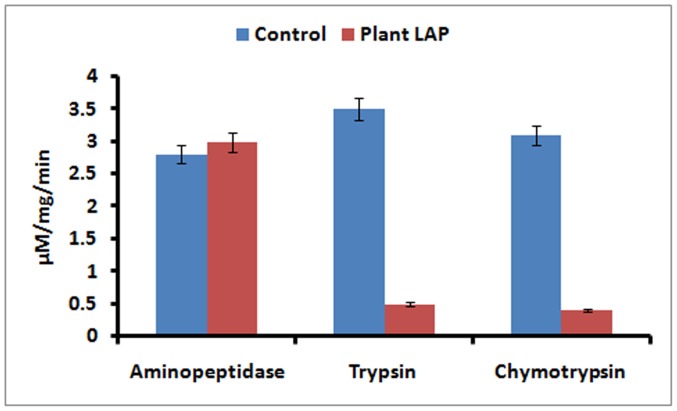
Determination of activities of aminopeptidase, trypsin and chymotrypsin in the midgut of *H. armigera* larvae fed on artificial diet and diet containing LAP. The spectrophotometric assay was carried out at 37°C using selective substrates viz. L*p*NA for aminopeptidase, BA*p*NA for trypsin and SAAPF*p*NA for chymotrypsin. Rate of liberated of *p*-nitroaniline in the reaction was measured at 410 nm. The experiment was performed three times with three biological replicates.

Aminopeptidase activity isoforms were visualized from the midgut extracts of larvae fed on each diet. Three prominent aminopeptidase activity isoforms were detected in the midgut extracts of larvae fed on artificial diet. These aminopeptidase activity isoforms were designated as AP1 to AP3. Among the three aminopeptidase activity isoforms AP1 and AP3 showed more activity as compared to AP2 (Figure 4A). Aminopeptidase activity isoforms showed similar pattern in the midgut of larvae fed on LAP supplemented diet as compared to artificial diet without LAP (Figure 4A). In case of serine proteinase, total ten proteinase activity isoforms were detected in the midgut extract of larvae reared on artificial diet. These *H. armigera* gut proteinase (HGP) isoforms were designated as HGP1 to HGP10 (Figure 4B). Interestingly, the activity of proteinase isoforms was significantly decreased in the midgut extracts of larvae reared on diet supplemented with pigeon pea LAP (Figure 4B). Activities of aminopeptidase, trypsin and chymotrypsin measured using selective chromogenic substrates. As observed in electrophoresis analysis aminopeptidase activities were found similar to the control in the midgut extracts of larvae reared on the diet containing plant LAP (Figure 5). However, trypsin and chymotrypsin activities were significantly decreased in the midgut extracts of larvae reared on diet supplemented with pigeon pea LAP (Figure 5).

### Interactions of Pigeon Pea Inducible LAP with *H.* *armigera* Gut Proteinases (HGPs)

**Figure 6:**
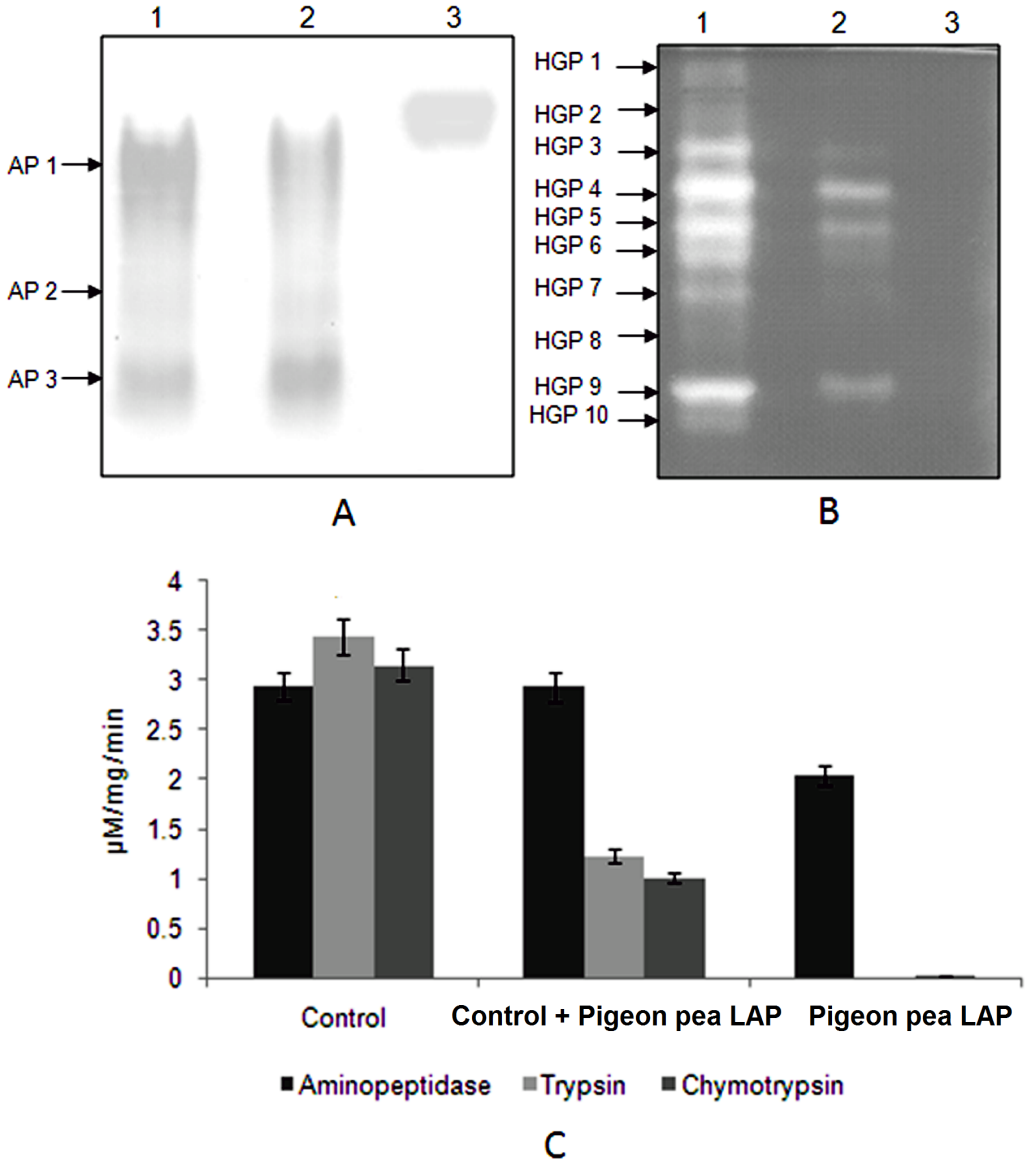
Interactions of pigeon pea inducible LAP with *H. armigera* gut proteinases (HGPs). To see the direct effect of inducible LAP on *H. armigera* gut serine protease activity present experiment was undertaken. A- Detection of aminopeptidase activity isoforms from the midgut extract of *H. armigera* by incubation with inducible LAP. Midgut extract (40 µg protein) of larvae fed on control diet was pre-incubated with pigeon pea LAP (40 µg protein) for at 37°C for 30 min and aminopeptidase activity isoforms in the mixture were electrophoretically visualized. Lane 1, crude midgut extract, lane 2, crude midgut extract+pigeon pea LAP and lane 3, pigeon pea LAP. B- Detection of proteinase activity isoforms in *H armigera* midgut extract when incubated with pigeon pea inducible LAP. Midgut extract (40 µg protein) of larvae fed on control diet was pre-incubated with pigeon pea LAP (40 µg protein) at 37°C for 30 min and proteinase activity isoforms in the mixture were visualized by gelatin reverse zymography. Lane 1, crude midgut extract, lane 2, crude midgut extract+pigeon pea LAP and lane 3, pigeon pea LAP.C- Determination of aminopeptidase, trypsin and chymotrypsin activities in *H armigera* midgut extract when incubated with pigeon pea inducible LAP. Midgut extract (40 µg protein) of larvae fed on control diet was pre-incubated with pigeon pea LAP (40 µg protein) at 37°C for 30 min and proteinase activities were determined by using selective chromogenic substrates such as L*p*NA, BA*p*NA and SAAPF*p*NA respectively for. The respective reactions were started at zero time and continued up 30 min (for aminopeptidase) and 1 h (for trypsin and chymotrypsin) and terminated by 30% acetic acid. The released *p*-nitroaniline from the substrates by the action of respective protease was measured at 410 nm. The assay was carried out in triplicate.

To evaluate direct effect of pigeon pea inducible LAP on *H. armigera* gut aminopeptidases and serine proteinases, the purified LAP was pre-incubated with the midgut extract of larvae reared on control diet and HGP activities were assessed using Zymogram analysis and enzyme assays. Results of zymography showed that *H. armigera* gut aminopeptidase activity was not affected by pigeon pea LAP (Figure 6A). However proteinase isoform activity was significantly reduced when the midgut extract was pre-incubated with plant LAP (Figure 6B). Similar results were observed in enzymes assays. The trypsin and chymotrypsin activities were significantly decreased in midgut extract pre-incubated with plant LAP. No change in the aminopeptidase activity was recorded in the midgut extract pre-incubated with plant LAP (Figure 6C).

### Substrate Specificity of *H. armigera* Aminopeptidase

The substrate specificity of aminopeptidases was evaluated from the midgut extracts of larvae fed on control and test diets using specific chromogenic peptide substrates. Results are summarized in Table 1. In the midgut extract of larvae reared on control diet, aminopeptidase activity was higher with L*p*NA as compared to A*p*NA. Negligible amount of aminopeptidase activity was recorded with V*p*NA. It was consistently observed that when aminopeptidase activity was found low with L*p*NA, in the same midgut extracts aminopeptidase activity was considerably higher with A*p*NA (Table 1).

**Table 1:** Substrate preferences of aminopeptidases of *H*. *armigera* fed diet with and without LAP.

Diet	Activity (U)		
	L*p*NA	A*p*NA	V*p*NA
Control	325.50±1.52	216.59±2.31	21.00±0.98
Plant LAP	324.74±1.87	229.20±2.81	20.50±0.92

1 unit (U) = one micromole of *p*-nitroaniline released/mg of protein/min at 37°C.

## Discussion

Aminopeptidases are the major N-terminal modifying enzymes which catalyze the hydrolysis of amino acids from the N-terminus of the proteins and peptides. These enzymes are ubiquitous in nature and their activities have been defined in animals, plants and prokaryotes [25]–[27]. Leucine aminopeptidase is considered as inducible component of defense in tomato [6] and involved in the late wound response and jasmonic acid perception [17]. It is interesting to find potential defensive functions of plant inducible aminopeptidase in the insect midgut. In the present paper, we report the effect of pigeon pea inducible LAP on the growth, survival and digestive physiology of *H. armigera* larvae. The *H. armigera* larvae reared on artificial diet without LAP showed normal growth. However, larval growth and survival rate was significantly reduced on the diet containing LAP. The function of plant LAP is still under discussion and it is likely that the different classes play various roles. For example, LAP acts as a transcriptional or posttranslational regulator of the jasmonic acid mediated signaling cascade and thus influences the overall defense response of the plant [14], [15].

Interestingly, in the present study we found that the serine proteinase activities were significantly decreased in the midgut of larvae reared on diet incorporated with pigeon pea LAP. Particularly, major digestive enzymes, trypsin and chymotrypsin activities were significantly reduced in the midgut of *H. armigera* larvae fed on diet containing plant LAP. Data support the hypothesis that plant inducible LAP may have direct defensive role in the insect midgut [9]. It is proposed that LAP have direct defensive function in the midgut of insect either by damaging it as shown from maize for cysteine protease [28], or by reducing the availability of free amino acids through liberation of arginine from peptides [9]. In this connection, we have studied direct *in vitro* interactions of pigeon pea LAP with *H. armigera* midgut aminopeptidases and serine proteinases. Qualitative and quantitative results from the present study suggest that *H. armigera* aminopeptidase activity was not affected by pigeon pea LAP. However the serine proteinase activity was significantly reduced when the midgut extract pre-incubated with LAP. The data indicate that pigeon pea LAP might be responsible for the degradation of insect serine proteinases which eventually leads to retarded growth and performance of the larvae.

Concentration of LAP (10 µg/g) for insect feeding was selected to maximize the likelihood of detecting an upper limit on the response. Plant defense compounds at their biological concentrations are capable of restricting insects. However previous feeding studies suggest that, the maximum adverse effect of plant defensive enzymes or other proteins only observed when they were either overexpressed or induced by artificial treatment (MeJA or wounding) or fed in higher concentrations. In a study Fowler et al., [17] measured the performance of *M. sexta* larvae on the tomato plants overexpressing LAP or LAP silenced or wild type. They found that LAP overexpressing tomato lines were more resistant while LAP silenced plants were susceptible to *M. sexta* feeding as compared to wild type plants. When the *S. nigrum* LAPs were downregulated using a transient virus-induced gene-silencing strategy [16], masses of *M. sexta* larvae were greater than insects that fed upon control plants. Unlike tomato, where LAP RNAs and proteins are usually absent or detected at low levels in healthy leaves, *S. nigrum* leaves accumulate substantial amounts LAP RNAs and proteins in non-damaged leaves [29]. The overexpression of polyphenol oxidase in tomato resulted in the increased resistance of plants against *Spodoptera litura* [30]. High concentrations of jasmonic and salicylic acid in artificial diet were found to be responsible for 5 to 7 fold increased expression of cytochrome P450 genes in *Helicoverpa zea* larvae [31]. Upon insect feeding plant has to induce defense cascades and overproduce defense proteins which ultimately become harmful and are responsible for reduced growth of the insects. Hence, it seems that the higher concentration of defense proteins (induced) is required to minimize the insect performance.

While evaluating substrate preferences, aminopeptidase activity was found higher with L*p*NA as compared to activity with A*p*NA in the midgut extract of larvae reared on diet without LAP. The results of differential substrate specificity of aminopeptidase correlate to that of previous findings [19], [32]. The obtained results point out that the pigeon pea inducible LAP may have direct role in the midgut of *H. armigera* in degradation of serine proteinase activity. Knowledge on the role of inducible plant LAPs in the insect midgut and their possible interactions with midgut soluble aminopeptidases and serine proteinases will be useful for planning strategies to control insect pests. The finding that pigeon pea inducible LAP reduces the performance of *H. armigera* larvae, illustrates that this phenomenon and category of enzymes holds potential for future studies with respect to plant-pest interactions.

## References

[1] Gatehouse JA (2002) Plant resistance towards insect herbivores: a dynamic interaction. New Phytol 156: 145–169. 10.1046/j.1469-8137.2002.00519.x 33873279

[2] Kessler A, Halitschke R, Baldwin IT (2004) Silencing the jasmonate cascade: induced plant defenses and insect populations. Science 305: 665–668. 15232071 10.1126/science.1096931

[3] Broadway RM (1995) Are insects resistant to plant proteinase inhibitors? J Insect Physiol 41: 107–116. 10.1016/s0022-1910(97)00028-0 12770497

[4] Jongsma MA, Bolter C (1997) The adaptation of insects to plant protease inhibitors. J Insect Physiol 43: 885–895. 12770458 10.1016/s0022-1910(97)00040-1

[5] Green TR, Ryan CA (1972) Wound-induced proteinase inhibitor in plant leaves: a possible defense mechanism against insects. Science 175: 776–777. 17836138 10.1126/science.175.4023.776

[6] Pautot V, Holzer FM, Reisch B, Walling LL (1993) Leucine aminopeptidase: an inducible component of defense response in *Lycopersicon esculentum* (tomato). Proc Natl Acad Sci U S A 90: 9906–9910. 8234334 10.1073/pnas.90.21.9906 PMC47681

[7] Constabel CP, Bergey DR, Ryan CA (1995) Systemin activates synthesis of wound-inducible tomato leaf polyphenol oxidase via the octadecanoid defense signaling pathway. Proc Natl Acad Sci U S A 92: 407–411. 7831300 10.1073/pnas.92.2.407 PMC42749

[8] Chao WS, Gu Y-Q, Pautot V, Bray EA, Walling LL (1999) Leucine aminopeptidase RNAs, proteins and activities increase in response to water deficit, salinity and the wound signals-systemin, methyl jasmonate, and abscisic acid. Plant Physiol 120: 979–992. 10444081 10.1104/pp.120.4.979 PMC59357

[9] Chen H, Wilkerson CG, Kuchar JA, Phinney BS, Howe GA (2005) Jasmonate-inducible plant enzymes degrade essential amino acids in the herbivore midgut. Proc Natl Acad Sci U S A 102: 19237–19242. 16357201 10.1073/pnas.0509026102 PMC1323180

[10] Broadway RM, Duffey SS (1986) Plant proteinase inhibitors: Mechanism of action and effect on the growth and digestive physiology of larval *Heliothis zea* and *Spodoptera exiqua* . J Insect Physiol 32: 827–833.

[11] Broadway RM, Duffey SS (1988) The effect of plant protein quality on insect digestive physiology and the toxicity of plant proteinase inhibitors. J Insect Physiol 34: 1111–1117.

[12] Duffey SS, Stout MJ (1996) Antinutritive and toxic components of plant defense against insects. Arch Insect Biochem Physiol 32: 3–37.

[13] Matsui M, Fowler JH, Walling LL (2006) Leucine aminopeptidases: diversity in structure and function. Biol Chem 387: 1536–1544. 10.1515/BC.2006.191 17132098

[14] Gu Y-Q, Holzer FM, Walling LL (1999) Overexpression, purification and biochemical characterization of the wound induced leucine aminopeptidase of tomato. Eur J Biochem 263: 726–735. 10469136 10.1046/j.1432-1327.1999.00548.x

[15] Walling LL (2006) Recycling or regulation? The role of amino-terminal modifying enzymes. Curr Opin Plant Biol 9: 227–233. 16597508 10.1016/j.pbi.2006.03.009

[16] Hartle M, Hogler M, Schmidt DD, Baldwin IT (2008) Optimized virus-induced gene silencing in *Solanum nigrum* reveals the defensive function of leucine aminopeptidase against herbivore and the shortcoming of empty vector controls. New Phytol 179: 356–365. 19086287 10.1111/j.1469-8137.2008.02479.x

[17] Fowler JH, Narváez-Vásquez J, Aromdee DN, Pautot V, Holzer FM, et al (2009) Leucine aminopeptidase regulates defense and wound signaling in tomato downstream of jasmonic acid. Plant Cell 21: 1239–1251. 19376935 10.1105/tpc.108.065029 PMC2685619

[18] Lomate PR, Hivrale VK (2011) Induction of leucine aminopeptidase (LAP) like activity with wounding and methyl jasmonate in pigeon pea (*Cajanas cajan*) suggests the role of these enzymes in plant defense in leguminosae. Plant Physiol Biochem 49: 609–616. 21420308 10.1016/j.plaphy.2011.02.023

[19] Lomate PR, Hivrale VK (2010) Partial purification and characterization of *Helicoverpa armigera* (Lepidoptera: Noctuidae) active aminopeptidase secreted in midgut. Comp Biochem Physiol B 155: 164–170. 19913106 10.1016/j.cbpb.2009.10.018

[20] Lowry OH, Rosebrough NJ, Farr AL, Randall RJ (1951) Protein measurement with the Folin phenol reagent. J Biol Chem 193: 453–464. 14907713

[21] Nagarkatti S, Prakash A (1974) Rearing *Helicoverpa armigera* (Hubner) on an artificial diet, Technical Bulletin, Vol. 17 Commonwealth Institute of Biological Control, Bangalore.

[22] Davis BJ (1964) Disc electrophoresis II. Methods and application to human serum. Ann NY Acad Sci 121: 404–427. 14240539 10.1111/j.1749-6632.1964.tb14213.x

[23] Bozic N, Vujcic Z (2005) Detection and quantification of leucyl aminopeptidase after native electrophoresis using leucine *p*-nitroanilide. Electrophoresis 26: 2476–2480. 15920779 10.1002/elps.200500047

[24] Feliocioli R, Garzelli B, Vaccari L, Melfi D, Balestreri E (1997) Activity staining of protein inhibitors of proteases on gelatin containing polyacrylamide gel electrophoresis. Anal Biochem 244: 176–178. 9025927 10.1006/abio.1996.9917

[25] McDonald JK, Barrett AJ (1986) Mammalian proteases. A glossary and bibliography. Vol 2, Academic Press, London.

[26] Rendueles PS, Wolf DH (1988) Proteinase function in yeast: biochemical and genetic approaches to a central mechanism of post-translational control in the eukaryotic cell. Fed Eur Microbiol Soc Microbiol Rev 54: 17–46. 10.1111/j.1574-6968.1988.tb02706.x-i1 3078768

[27] Walling LL, Gu Y-Q (1996) Plant aminopeptidase: Occurrence, function and characterization. In: Taylor A, (Eds.), Aminopeptidase. R.G. Landes, Georgetown, Texas, 174–219.

[28] Pechan T, Cohen A, Williams WP, Luthe DS (2002) Insect feeding mobilizes a unique plant defense protease that disrupts the peritrophic matrix of caterpillars. Proc Natl Acad Sci U S A 99: 13319–13323. 12235370 10.1073/pnas.202224899 PMC130631

[29] Chao WS, Pautot V, Holzer FM, Walling LL (2000) Leucine aminopeptidases: the ubiquity of LAP-N and the specificity of LAP-A. Planta 210: 563–573. 10787049 10.1007/s004250050045

[30] Mahanil S, Attajarusit J, Stout M, Thipyapong P (2008) Overexpression of tomato polyphenol oxidase increases resistance to common cutworm. Plant Sci. 174, 456–466.

[31] Li X, Schuler M, Berenbaum M (2002) Jasmonate and salicylate induce expression of herbivore cytochrome P450 genes. Nature 419: 712–715. 12384696 10.1038/nature01003

[32] Bozic N, Vujcic Z, Nenadovic V, Ivanovic J (2003) Partial purification and characterization of midgut leucyl aminopeptidase of *Morimus funereus* (Coleoptera: Cerambycidae) larvae. Comp Biochem Physiol 134B: 231–241. 10.1016/s1096-4959(02)00257-9 12568801

